# Experimental Analysis of Aerodynamic Loads of Three-Bladed Rotor

**DOI:** 10.3390/ma15093335

**Published:** 2022-05-06

**Authors:** Zofia Szmit, Lukasz Kloda, Marcin Kowalczuk, Grzegorz Stachyra, Jerzy Warmiński

**Affiliations:** Department of Applied Mechanics, Faculty of Mechanical Engineering, Lublin University of Technology, 20-618 Lublin, Poland; l.kloda@pollub.pl (L.K.); m.kowalczuk@pollub.pl (M.K.); g.stachyra@pollub.pl (G.S.); j.warminski@pollub.pl (J.W.)

**Keywords:** rotating beams, aerodynamic loads, synchronization

## Abstract

The purpose of the current study is the experimental analysis of the dynamics and aerodynamic loads of a three-bladed rotor. The experimental tests focus on the rotation with three different angular velocities; for each angular speed, four different preset angles of beam have been studied. During the laboratory experiment, strain gauges, as well as high-speed cameras, have been used as the measurement system. The images from the high-speed cameras have been used to obtain aerodynamic loads in the form of polynomials, while the signals from strain gauges mounted on each beam allowed us to observe the synchronization phenomenon between beams.

## 1. Introduction

Rotating structures are very important and popular in mechanical engineering, and they are used widely in several industrial applications. The most popular rotating components are helicopter rotors, fans, airplane propellers, turbojet aircraft engines, wind turbines, etc. Recently, many of these elements have been made of composite materials, because they have many technological advances, such as better strength properties, high stiffness, and a better strength-to-weight ratio.

The in-plane free vibrations analysis of rotating beams is considered in [1], where the authors used an exact dynamic stiffness method. Furthermore, in the calculation, the Coriolis effects, as well as the effects of an arbitrary hub radius, are taken into account. The governing equations of motion have been derived from Hamilton’s principle, considering the stiffness matrix depending on a beam’s frequency. The free vibrations analysis is studied also in [2]—the authors focused on a rotating hub with a functionally graded material beam system. The equations of motion are obtained from the Lagrange equation of the second kind, while the model is based on the complex dynamics of a rigid body and flexible beam theory. Moreover, Oh et al. [3] studied a rotating system with a hub and FGM beam, where the equations of motion are derived from the Rayleigh–Ritz assumed mode method with Kane’s method. The authors analyzed the dynamics of a pretwisted beam and showed the effects of the Young’s modulus ratio, hub radius ratio, pre-twist angle, taper ratios, width-to-thickness ratio, and angular speed upon the dimensionless natural frequencies of the FGM blade. Additionally, they presented an algebraic condition under which natural frequencies can be minimized. The geometrically exact model of a rotating isotopic blade is proposed in [4]. In this paper, the authors focused on the linear modal properties of the blade, while investigating natural frequencies, as well as coupling between flapping, lagging, axial, and torsional components. Furthermore, the nonlinear free vibrations of a centrifugally stiffened beam with a uniform cross-section and constant angular velocity are analyzed in [5]. The authors used geometrically exact equations of motion for a Timoshenko beam that exhibits a large amplitude of displacement. The set of equations is derived from Hamilton’s principle and the model takes into account the Coriolis effect as well as warping of the cross-section. Based on this, the authors plotted backbone curves and analyzed the change in the behavior of the beam in the first lagging and flapping modes. The model of the pre-twisted composite rotor blade is presented in [6]. Shang et al. derived the geometrically exact equations of motion based on Hamilton’s principle for a Timoshenko beam with an arbitrary cross-section. The analytical results of the static and dynamic analysis have been compared with experimental data. The authors focused mainly on the influence of the transverse shear deformation on the analyzed pre-twisted blade. Sun et al. presented a dynamic model of a multilayer rotating beam mounted on an arbitrary stagger angle in [7]. They presented simulations for reduced 2D and full 3D models, which show excellent agreement. They also analyzed the change in the damping in rotation of the different angular velocities. Moreover, a rotating thin-walled cylindrical shell is presented in [8]. The authors used the Lagrange equation to obtain equations of motion; next, they analyzed the nonlinear vibrations of the model, and the influence of the angular speed and geometry parameters of the shell on system dynamics. The nonlinear resonances of a rotating axially functionally graded beam are presented in [9]. In the governing equations, axial, chordwise, and flapwise deformations are taken into account. The analytical results of calculations have been compared with results based on the finite element method. The authors concluded that the analyzed model of the beam exhibited complicated nonlinear behavior. The dynamics of the rotating hub–beam system are presented in [10,11], in which a model of a slender beam with a tip mass based on Bernoulli–Euler theory is developed. The equations of motion are derived from Hamilton’s principle, and then partial differential equations are solved analytically with the multiple time scale method. The forced vibrations, the influence of beam rotation, and the tip mass are studied. The model of the hub and the thin-walled composite beam is presented in [12]. The authors analyzed not only the dynamics and the natural and forced vibrations of the system, but also developed the control methods of the rotating structures. The embedded active elements are used to control the oscillations of the model. Moreover, the model of the rotating hub–beam system based on Timoshenko beam theory is developed in [13] with equations of motion derived from Hamilton’s principle. Non-classical effects such as material anisotropy, transverse shear, and primary and secondary cross-section warpings are taken into account in the equations of motion. Latalski et al., in [14], obtained a model of a rotating hub with a thin elastic clamped bimorph beam. Two layers of active elements, which play the role of a sensor/actuator, are applied in the model. The authors showed the results of numerical simulations of the model excited by a periodic torque. The concept and development of the three-blade rotor model is shown in [15]. The authors presented numerical analysis based on the finite element method, as well as series of static and dynamic experimental simulations. In the case of both methods, good agreement between results is observed. The dynamics of the system composed of the rigid hub and three composite beams is analyzed in [16,17]. Forced vibrations of the model and the synchronization phenomenon, which can be observed between beams or between beams and the hub, are studied in detail. Two types of excitations of the rotor are analyzed—a regular torque or chaotic oscillations. Experimental tests of the helicopter-like thin-walled rotating beam are presented in [18]. The tests have been carried out in a vacuum chamber, where the blades have been excited by impulse force. During the experiment, time series as well as strain distribution have been recorded. Two other experimental approaches using piezoelectric actuators together with a contactless scanning vibrometer and modal hammer were presented by Teter and Gawryluk in [19]. Their laboratory tests were extended by a numerical finite element model; however, a cantilever beam, one-beam–hub, and three-beam–hub structures were not put in rotational motion. Additionally, modal analysis of the thin-walled beam is shown in [20], in which the authors compare theoretical and experimental results for a composite box beam. During the experimental tests, macro fiber composite (MFC) patch actuators were used for the excitation of the model and the laser-based motion analysis system functioned as the recorder. Furthermore, the results of numerical calculations and experimental tests of the three-bladed rotor are presented in [21]. The experimental studies of the rotating beam are also presented in [22], in which the authors tested three different beams rotating with a constant angular velocity. The results from experimental studies are compared with results from finite element method simulations. Observations of the constant angular velocity of the three-bladed hub with simultaneous harmonic excitation via piezoelectric patches—called active control—are presented in [23], and the corresponding numerical experiment is presented in [24].

In the present contribution, experimental studies on the three-bladed rotor are introduced. The rotor under investigation consists of three slender and elastic beams attached to the rigid hub. During the laboratory tests, the angular speed of the hub, as well as the preset angle, have been changed. For each angular velocity (50, 100, and 150 rpm), four different preset angles have been set ($0^{\circ }$, $5^{\circ }$, $45^{\circ }$, and $90^{\circ }$). Two types of measurements have been used—two high-speed cameras and three strain gauges. The cameras have been used for optical contactless measurements, while strain gauges embedded on each beam allowed us to record time series during the beam deformations and rotation of the system. Additionally, based on time series, the synchronization phenomenon is analyzed.

## 2. Model and Experimental Methods

**Figure 1:**
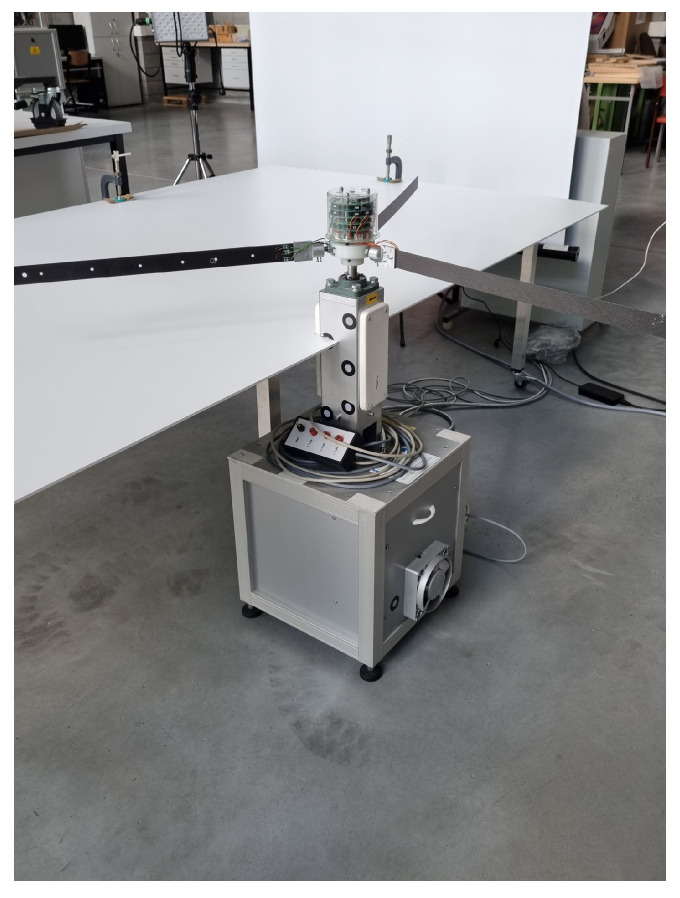
Three-bladed rotor—laboratory stand.

The experimental tests have been performed in the Laboratory of the Department of Applied Mechanics of Lublin University of Technology. The laboratory stand is shown in Figure 1. The studied rotor is composed of three flexible composite beams and a rotating rigid hub. All of the blades are composed of an eighteen-layered laminate of graphite-epoxy prepreg material, ThinPregTM 120EP-513/CF resin reinforced with M40JB-12000-50B TORAY carbon fiber composite [12], which is applied in the stacking sequence ($0^{\circ }$/$-60^{\circ }$/$60^{\circ }$/$0^{\circ }$/$-60^{\circ }$/$60_{3}^{\circ }$/$-60_{2}^{\circ }$/$0_{2}^{\circ }$/$-60^{\circ }$/$0_{2}^{\circ }$/$60_{2}^{\circ }$/$-60^{\circ }$) [25]. Each single beam has a length of 595 mm, with a cross-section 34×0.9 mm and density ρ=1350 kg/m${}^{3}$. Near the fixed end of each beam, 25 mm from the dedicated mount, the strain gauge (model TFr-8/120) is embedded. In order to measure strain not only in the longitudinal direction, but also torsion, a rosette strain gauge (model TFr-8/120) is used, with the main part of the sensor positioned along the span of the beam in its axis, and two remaining parts in ±$60^{\circ }$ from the main axis of the beam. The strain gauge’s grid material is constantan foil and the backing material is modified epoxy-phenolic resin. Nominal resistance of the sensor is 120 Ω and strain sensitivity k=2.1 for each strain gauge.

The signals from the strain gauges are sent to computer software by the wireless module. The radius of the rigid body hub is 120 mm. In the hub, there are boards with electronic circuits with the wireless module, which allows us to transmit signals from the beams. The laboratory set-up is excited by a DC motor with power of 1.1 kW (1.5 HP), and the motor is combined with the hub by a helical clutch. Between the hub and the motor, a set of nine slip rings is applied—the rings allow us to transmit measuring and control signals. The test stand was designed for scientific research and experiments with constant velocity or excitation of any optional signal, as well as excitation with different frequencies. In the authors’ previous paper [21], the experimental tests of the rotor excited by different frequencies, as well as the resonance curvature, are presented.

**Figure 2:**
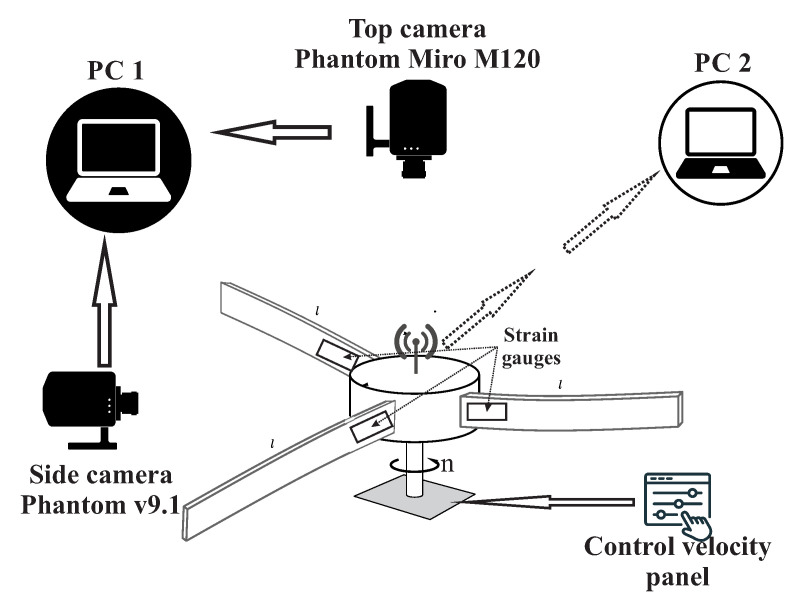
Diagram of the measuring system.

This contribution is focused on experiments with a constant angular velocity. During the tests, two parameters have been changed: the angular velocity and the preset angle of the blade. The authors considered four different setting angles: $0^{\circ }$, $5^{\circ }$, $45^{\circ }$, and $90^{\circ }$. For every angle, three different angular velocities of the rotor have been set: 50 rpm, 100 rpm, and 150 rpm. Two high-speed cameras have been used, as well as signals from strain gauges in the measurement setup; see Figure 2. One camera was hung on the top of the rotor to capture the view of the whole stand, while the second camera was placed perpendicularly to the rotation axis. In order to cover the whole motion range, the top camera was placed 2.5 m away from the rotation plane.

The camera above the rotor was the Phantom Miro M120, which is based on a 2 Mpx sensor and 1.6 Gpx/s throughput, which translates to over 1200 frames per second at 1152×1152 resolution. The lens was selected for the exposure time and the area of the rotor, as well as the distance from the camera to the object intended to be captured. The best lens available was the Nikon AF 20 mm f/2.8 D; the shutter was set to the maximal settings. Since the resolution was set to 1216×1200 pixels, the maximal sample rate available was 1100 fps, and all the tested velocities were sampled with this rate. The side camera was set in a manner so as to cover only one full length of a beam with part of a hub. For the side view, the Phantom v9.1 was used, with a built-in CMOS sensor, which provides up to 1000 fps at full resolution of 1632×1200 active pixels. The lens selected for this angle was the Nikon AF 50 mm f/1.4 D. In order to visualize the entire motion of the beam, the desired resolution was 1244×720 pixels and the sample rate was set to 1900 fps for all tests.

## 3. Results—Video Measurements

**Figure 3:**
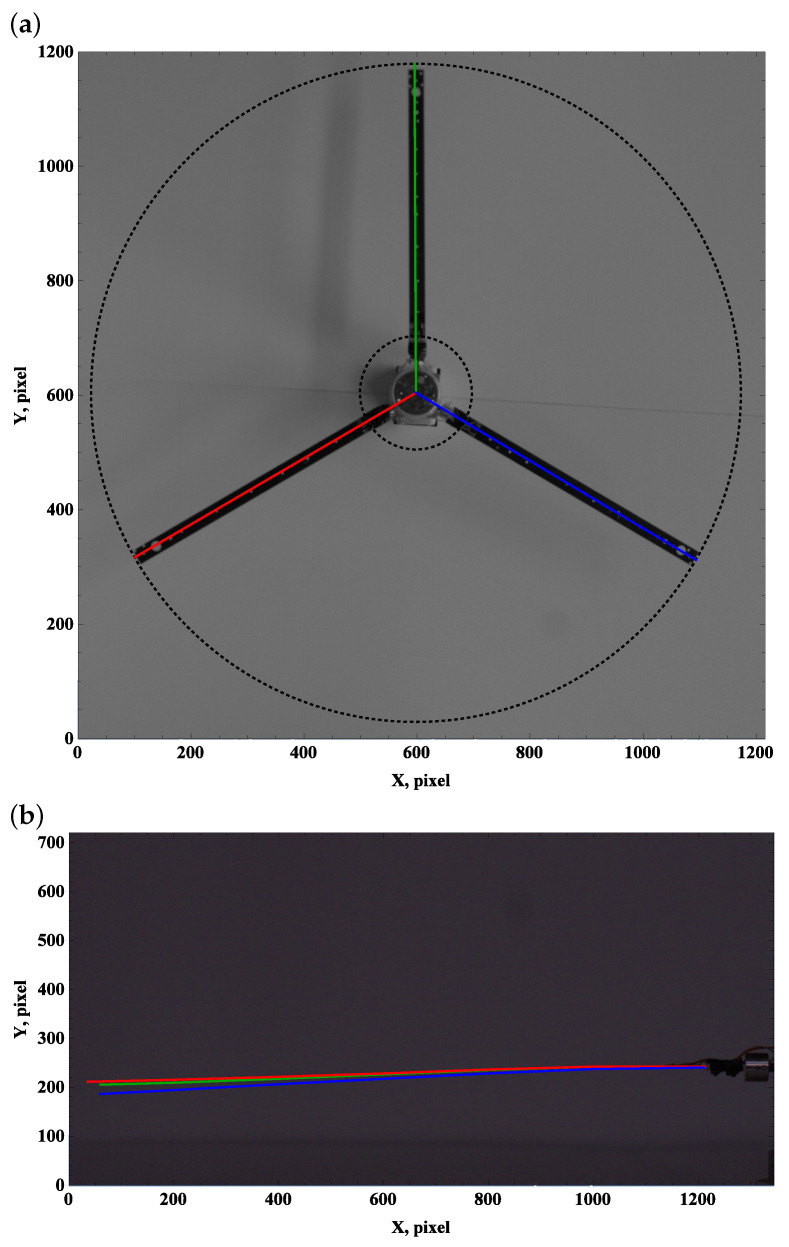
Preset angle $0^{\circ }$, angular speed 50 rpm: top view (**a**), side view (**b**).

**Figure 4:**
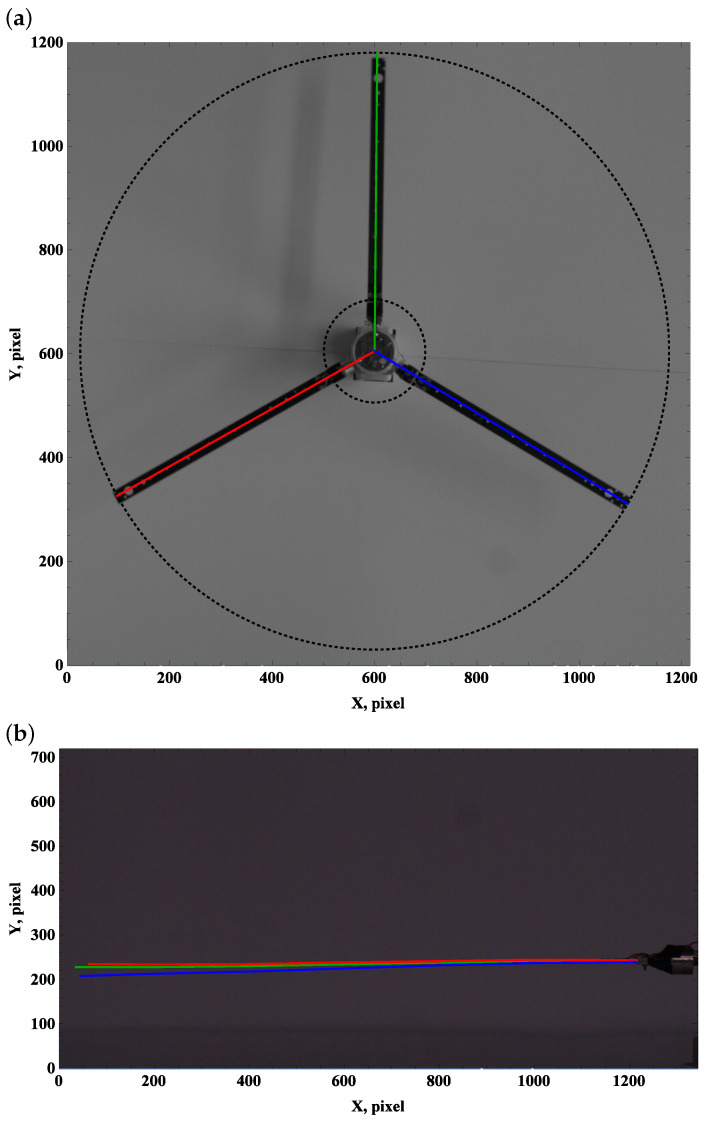
Preset angle $0^{0}$, angular speed 100 rpm: top view (**a**), side view (**b**).

**Figure 5:**
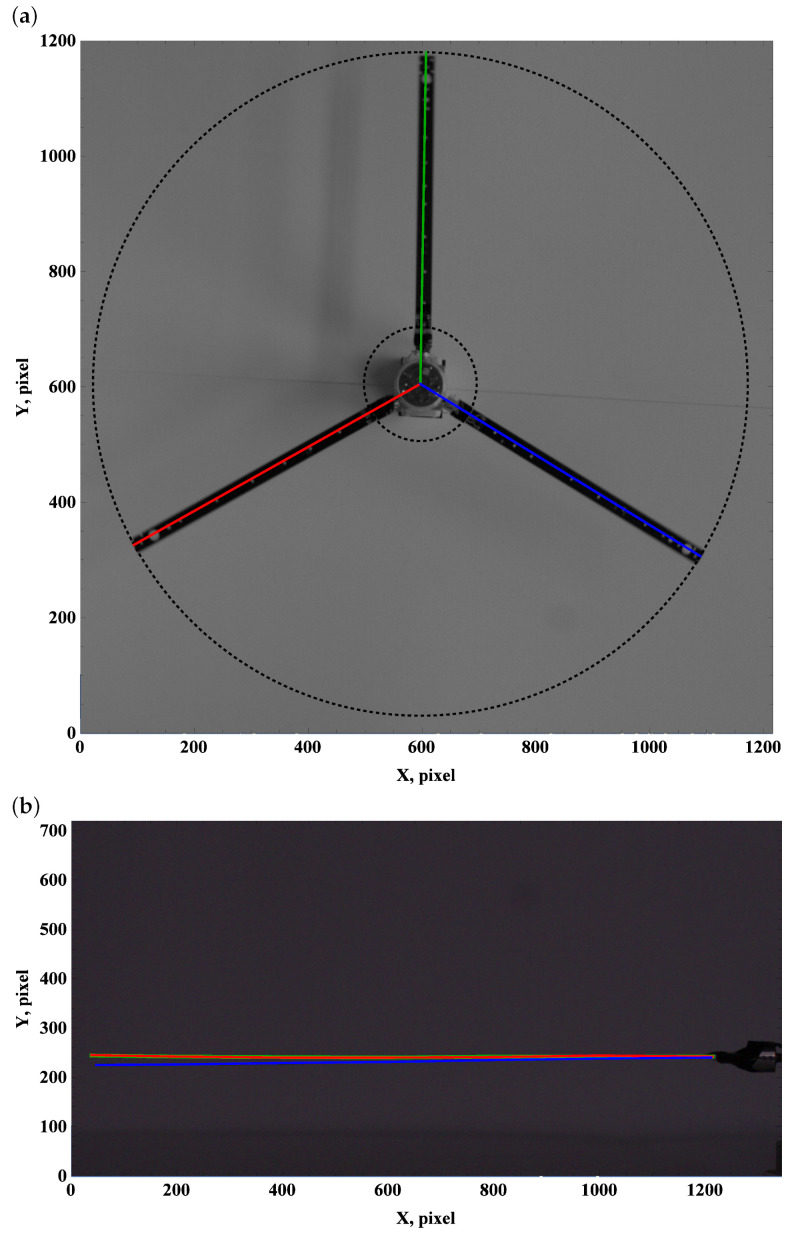
Preset angle $0^{\circ }$, angular speed 150 rpm: top view (**a**), side view (**b**).

**Figure 6:**
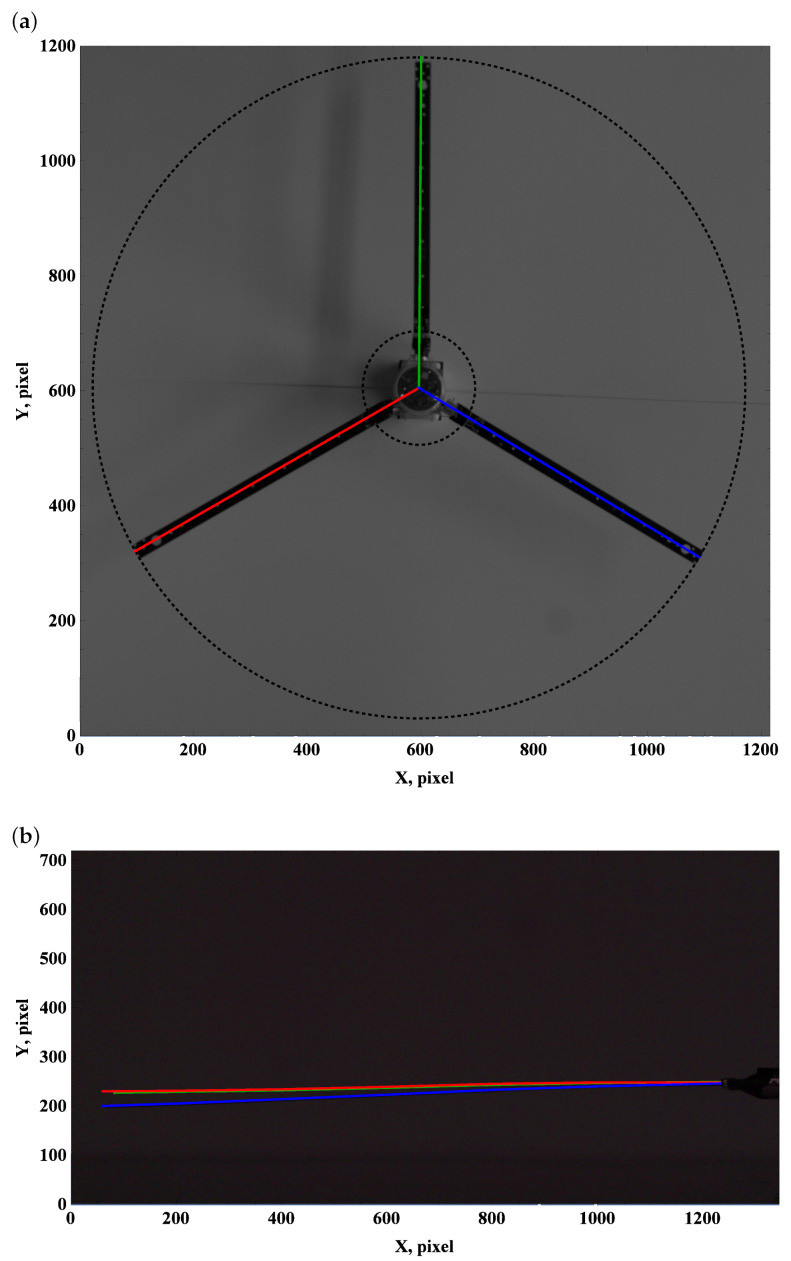
Preset angle $5^{\circ }$, angular speed 50 rpm: top view (**a**), side view (**b**).

**Figure 7:**
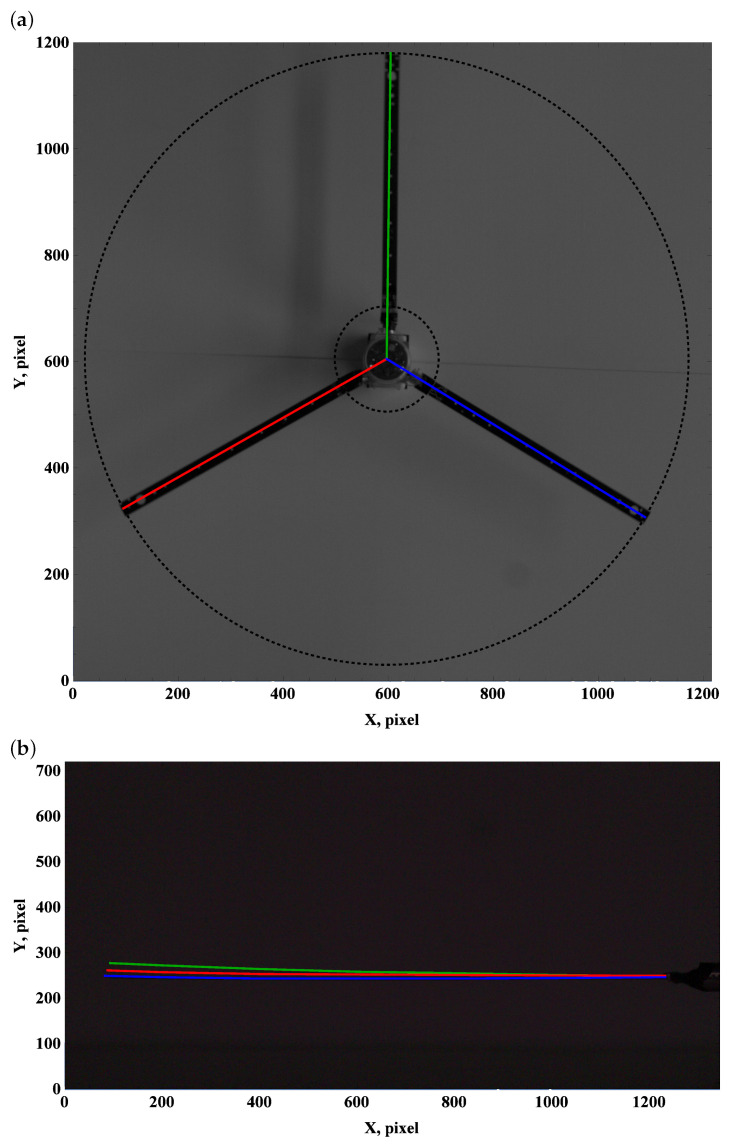
Preset angle $5^{\circ }$, angular speed 100 rpm: top view (**a**), side view (**b**).

**Figure 8:**
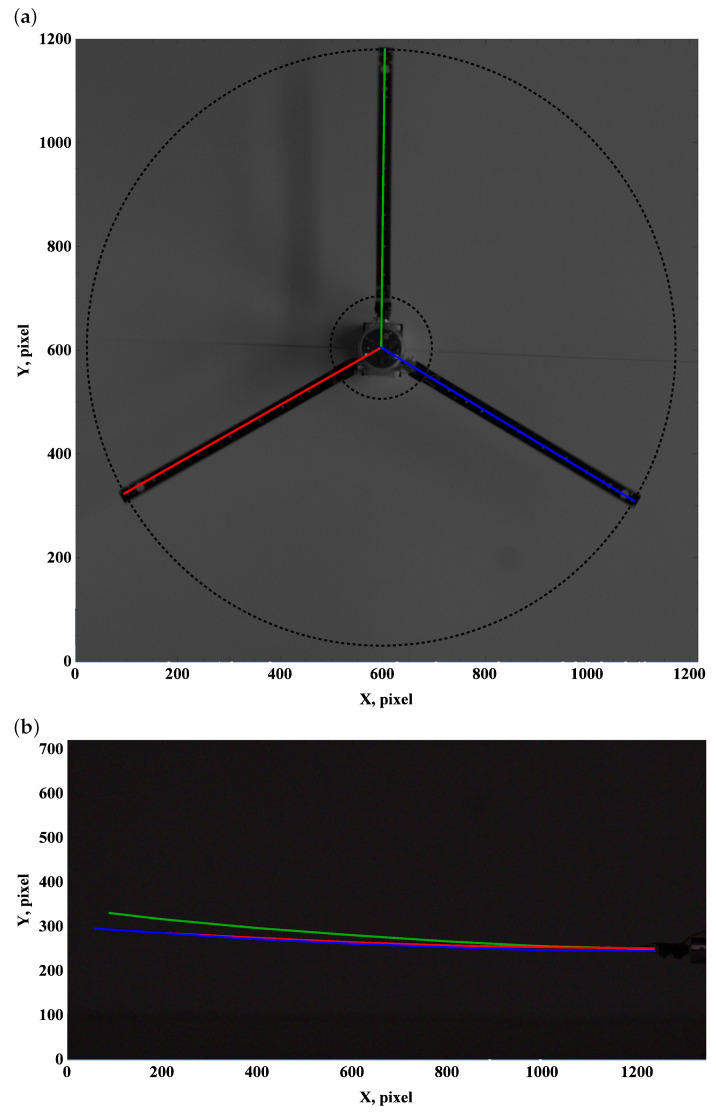
Preset angle $5^{\circ }$, angular speed 150 rpm: top view (**a**), side view (**b**).

**Figure 9:**
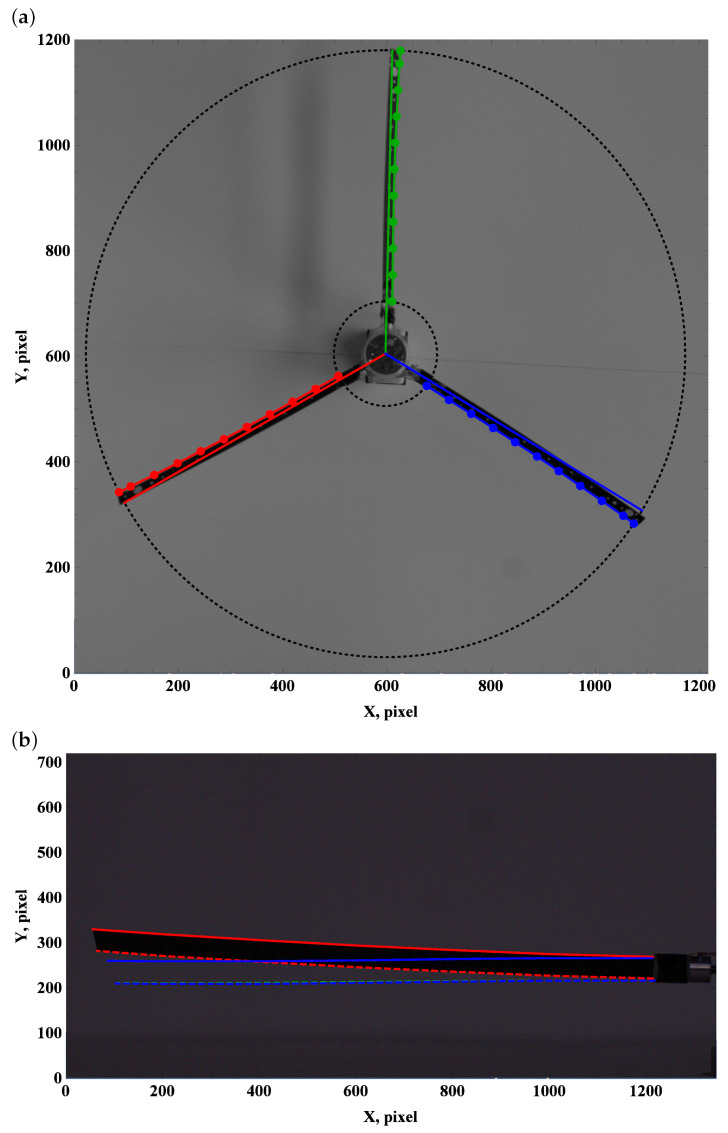
Preset angle $45^{\circ }$, angular speed 50 rpm: top view (**a**), side view (**b**).

**Figure 10:**
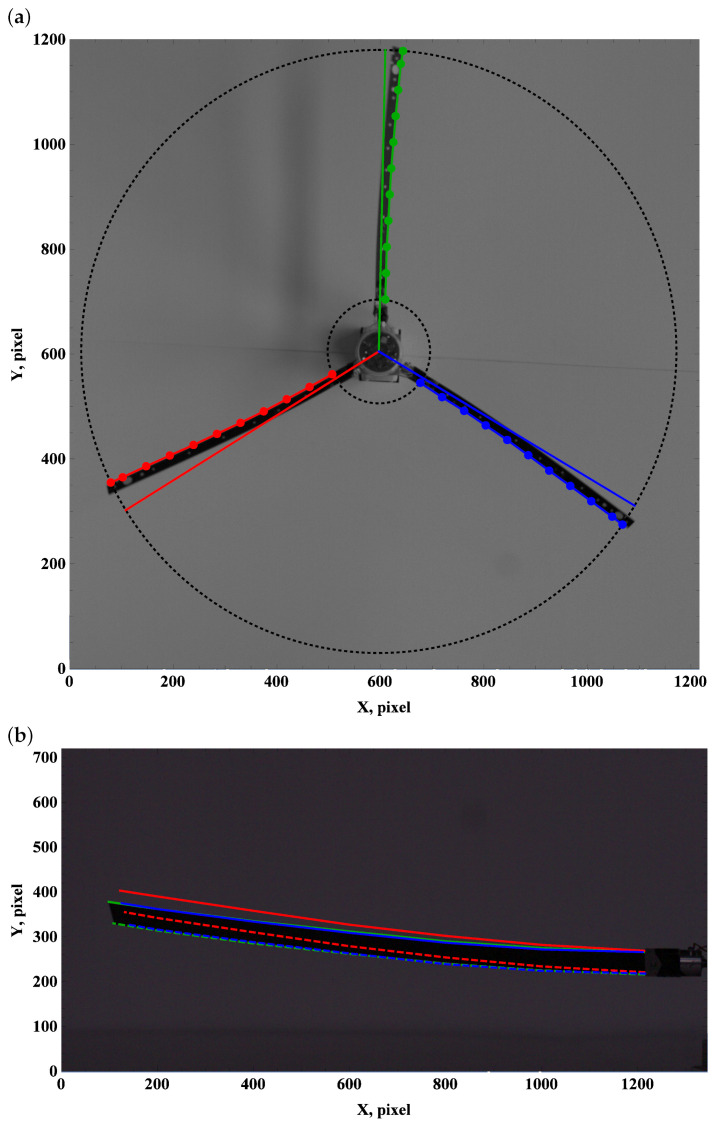
Preset angle $45^{\circ }$, angular speed 100 rpm: top view (**a**), side view (**b**).

**Figure 11:**
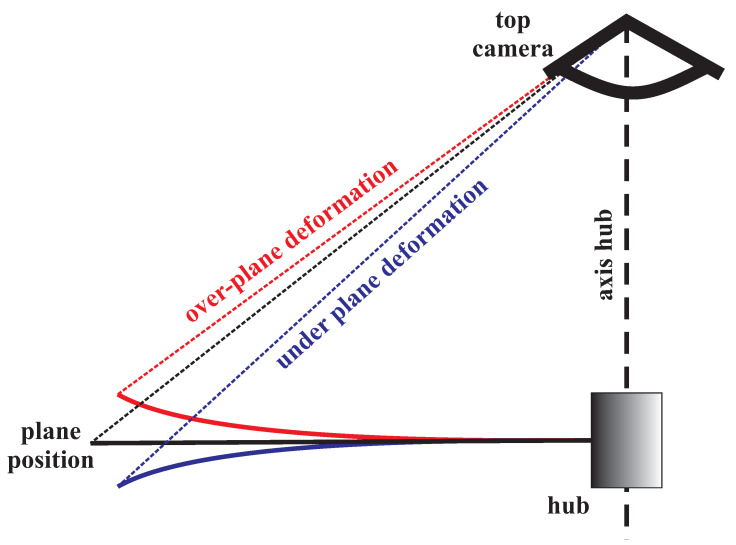
Side view of the top camera axially aligned with rotating hub: the effect of beam deformation outside the rotor plane on camera observation.

**Figure 12:**
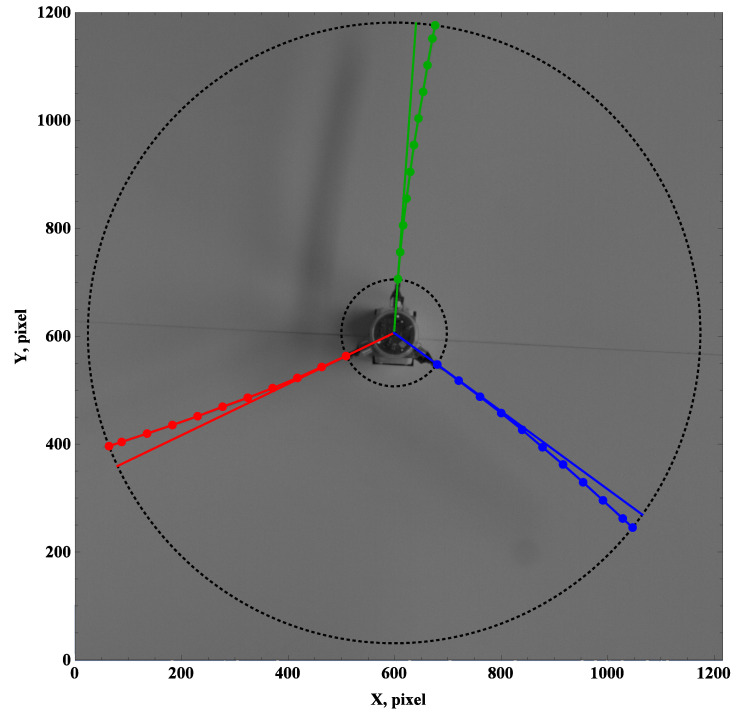
Preset angle $90^{\circ }$, angular speed 50 rpm: top view; side view is not applicable.

The videos obtained from the two high-speed cameras were post-processed in Mathematica commercial software. Figure 3, Figure 4, Figure 5, Figure 6, Figure 7, Figure 8, Figure 9, Figure 10, Figure 11 and Figure 12 are elaborated on the basis of single pixels for a selected freeze-frame at the highest deformations of the beam; we then placed them in a planar coordinate system and then construction lines and points were plotted on them. The results of this painstaking manual work are grouped according to the preset angle ($0^{\circ }$, $5^{\circ }$, $45^{\circ }$, and $90^{\circ }$) in Section 3.1, Section 3.2, Section 3.3 and Section 3.4, and ordered from the lowest to the highest angular speed (50, 100, 150 rpm). Note that the greater the preset angle of the beam, the greater the aerodynamic drag. Therefore, measurements for the preset angle $45^{\circ }$ and angular velocity greater than 100 rpm, as well as the preset angle $90^{\circ }$ and angular velocity greater than 45 rpm, are above the drive’s operating range.

We introduce colors green, red, and blue to construct lines for beams A, B, and C, respectively. Considering the top view frames, straight lines originate from the hub axis $0_{1}$ = ($X_{0}$,$Y_{0}$) and reflect the longitudinal axis of the undeformed beam. Line segments are described by origin $0_{1}$ and tip points defined at a distance $R_{2}$ from the origin and angle from the reference direction (horizontal edge of the photo, positively defined right direction) $\Phi_{i}$, where i=1,2,3.

(1) $$\begin{array}{c} X_{At}=X_{0}+R_{2}\cos\Phi_{1},\;Y_{At}=Y_{0}+R_{2}\sin\Phi_{1},\\ X_{Bt}=X_{0}+R_{2}\cos\Phi_{2},\;Y_{Bt}=Y_{0}+R_{2}\sin\Phi_{2},\\ X_{Ct}=X_{0}+R_{2}\cos\Phi_{3},\;Y_{Ct}=Y_{0}+R_{2}\sin\Phi_{3}. \end{array}$$

In the case of a perfect (rigid) anchorage of the beam in the hub, the relationship between the angles is $\Phi_{2}=\Phi_{1}+120^{\circ }$ and $\Phi_{3}=\Phi_{2}+120^{\circ }$. The blade grip is mounted in the polyamide ring, so the connection has the required stiffness and the processing requires corrections

(2) $$\Phi_{2}=\Phi_{1}+120^{\circ }+\alpha_{1},\;\Phi_{3}=\Phi_{1}+240^{\circ }+\alpha_{2}.$$

Coefficients $\alpha_{1}$ and $\alpha_{2}$ can vary $\pm 1^{\circ }$. Two black dotted circles are drawn in the top view pictures, which describe the radii responsible for the fixed edge $R_{1}=100$ pixels and free end $R_{2}=575$ pixels of the straight beam (no gravity, no centrifugal forces, and no aerodynamic forces). The outer circle indicates the reduction in the dimension due to deflection by gravity, centrifugal forces, and aerodynamic forces. An important effect reveals when the beam leaves the rotor plane. A lift towards the camera gives the optical effect of the beam extension, while moving away from the camera shortens its length; see Figure 11.

The side view frames are labeled with three curvilinear lines describing the deformation of the beam at a given moment. It requires comment. These side view pictures were not taken at the maximum amplitude of the swing out of rotor plane, but they were taken randomly. Capturing an image of the maximum vibration amplitude in a given plane is very demanding, beyond the capabilities of the equipment used. The situation would be different with a camera following the rotor and recording the full movement of the blade, which, of course, generates many more technical issues.

### 3.1. Preset Angle $0^{\circ }$

The zero preset angle almost does not generate lift, and drag force can be neglected. This implies that the beam deformation field undergoes a balance of gravity and centrifugal force. The top view, which is used to measure deformation due to drag and centrifugal forces, remains unchanged; see Figure 3a, Figure 4a and Figure 5a.

Carefully comparing the graphics, one can observe how the tip of the beam approaches and then crosses the outer circle as the angular velocity increases. This is in line with the side view. As the angular velocity gradually grows, the centrifugal force dominates over gravity, and the beam becomes almost perfectly straight; see Figure 3b, Figure 4b and Figure 5b.

### 3.2. Preset Angle $5^{\circ }$

A positive preset angle of approximately 5 degrees effectively generates lift and drag forces. However, aerodynamic forces have no significant effect on the top view frames (Figure 6a, Figure 7a and Figure 8a). The lift force generates the lifting of the beam end. Angular velocity of approximately 50 rpm is enough to balance gravity, and higher angular velocities of 100 rpm and 150 rpm bend the beam upwards; see Figure 6b, Figure 7b and Figure 8b.

### 3.3. Preset Angle $45^{\circ }$

Unlike the previous cases, the preset angle of $45^{\circ }$ changes the deformation field in both views. For the top views (Figure 9a, Figure 10a, Figure 11a and Figure 12a), the beam trailing edges are marked by 11 points and linked by a curve. In the next section, markers will be used to build a deformation profile and its polynomial approximation.

In Figure 9a,b, the leading (trailing) edges are marked with solid (dashed) lines. Differences in curves indicate a twist effect or, more likely, another optical issue. However, the graphical results can be used to describe the beam deformation field, and the narrow/detailed oscillations will be analyzed in a future section using the time histories of the strain gauges. Herein, a hypothesis can be accepted that the deformation profiles can be assigned to the axis offset in the upcoming measurements performed by the strain gauges.

### 3.4. Preset Angle $90^{\circ }$

In this case, the direction of high flexural stiffness prevents deformations out of rotor plane in video analysis; thus, the side view is not reported here. On the contrary, the direction susceptible to bending is subjected to supreme drag, which results in a very large deformation curve at angular velocity 50 rpm. Attempts to measure higher speeds (higher than 80 rpm) have been neglected due to the limited power of the DC motor.

## 4. Mathematical Description of Deformation Field

In top view camera records, three measurements are enough to collect information about beam deformation: two for preset angle $45^{\circ }$ and one for $90^{\circ }$. The measuring points have been divided into 11 radii values; more precisely, $r_{1}$–$r_{10}$ are spaced from each other every 50 pixels starting from $R_{1}$, and plus $r_{11}=R_{2}$. The radii values define the circles on which the angular value $\phi_{A1}$–$\phi_{A11}$ of the deflection with respect to the undeformed line segment $\Phi_{1}$

(3) $$\begin{array}{c} P_{Ai}\left(X_{Ai},Y_{Ai}\right)=\left(X_{0}+r_{i}\cos\left(\Phi_{1}+\phi_{Ai}\right),Y_{0}+r_{i}\sin\left(\Phi_{1}+\phi_{Ai}\right)\right), \end{array}$$

(4) $$\begin{array}{c} P_{Bi}\left(X_{Bi},Y_{Bi}\right)=\left(X_{0}+r_{i}\cos\left(\Phi_{2}+\phi_{Bi}\right),Y_{0}+r_{i}\sin\left(\Phi_{2}+\phi_{Bi}\right)\right), \end{array}$$

(5) $$\begin{array}{c} P_{Ci}\left(X_{Ci},Y_{Ci}\right)=\left(X_{0}+r_{i}\cos\left(\Phi_{3}+\phi_{Ci}\right),Y_{0}+r_{i}\sin\left(\Phi_{3}+\phi_{Ci}\right)\right),\\ \mathrm{where}\;i\in N=\{1,2,3,...\;11\}. \end{array}$$

The distance between the given point and constructing a line segment is obtained with a system of algebraic equations:

(6) $$\begin{array}{c} Y_{0}=c_{A1}X_{0}+c_{A2},\;Y_{At}=c_{A1}X_{At}+c_{A2},\;\to c_{A1},c_{A2}, \end{array}$$

(7) $$\begin{array}{c} d_{Ai}=\frac{c_{A1}X_{Ai}-Y_{Ai}+c_{A2}}{\sqrt{X_{Ai}^{2}+Y_{Ai}^{2}}}, \end{array}$$

where coefficients $c_{A1}$ and $c_{A2}$ describe the equation of a line intersecting the points ($X_{0}$,$Y_{0}$) and ($X_{AT}$,$Y_{AT}$); $d_{Ai}$ is the dimension (in pixels) between the line and the *i*th point. The authors introduce the dimensionless results in reference to the beam length; thus, relative deformation becomes $d_{Ai}/\left(R_{2}-R_{1}\right)$. Note that the beam length is a constant parameter $R_{2}-R_{1}$ of the initially straight (undeformed) beam; of course, the top and side views from cameras display different values in pixels, but still it is the same length ($L=R_{2}-R1$). Thus, in order to unify the results, the following projections of beam deformation on the proper plane (relative deformation) are presented for a dimensionless quantity of the beam length.

**Figure 13:**
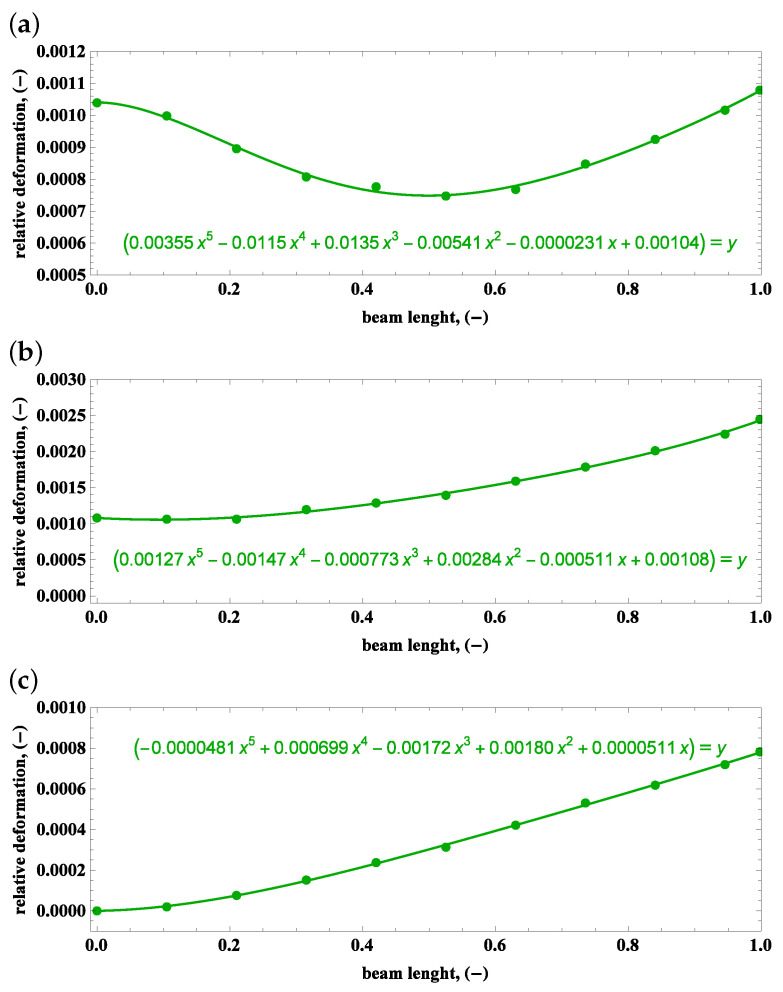
Beam deformations and their description by polynomials: (**a**) preset angle $45^{\circ }$ and angular speed 50 rpm, (**b**) preset angle $45^{\circ }$ and angular speed 100 rpm, (**c**) preset angle $90^{\circ }$ and angular speed 50 rpm.

**Figure 14:**
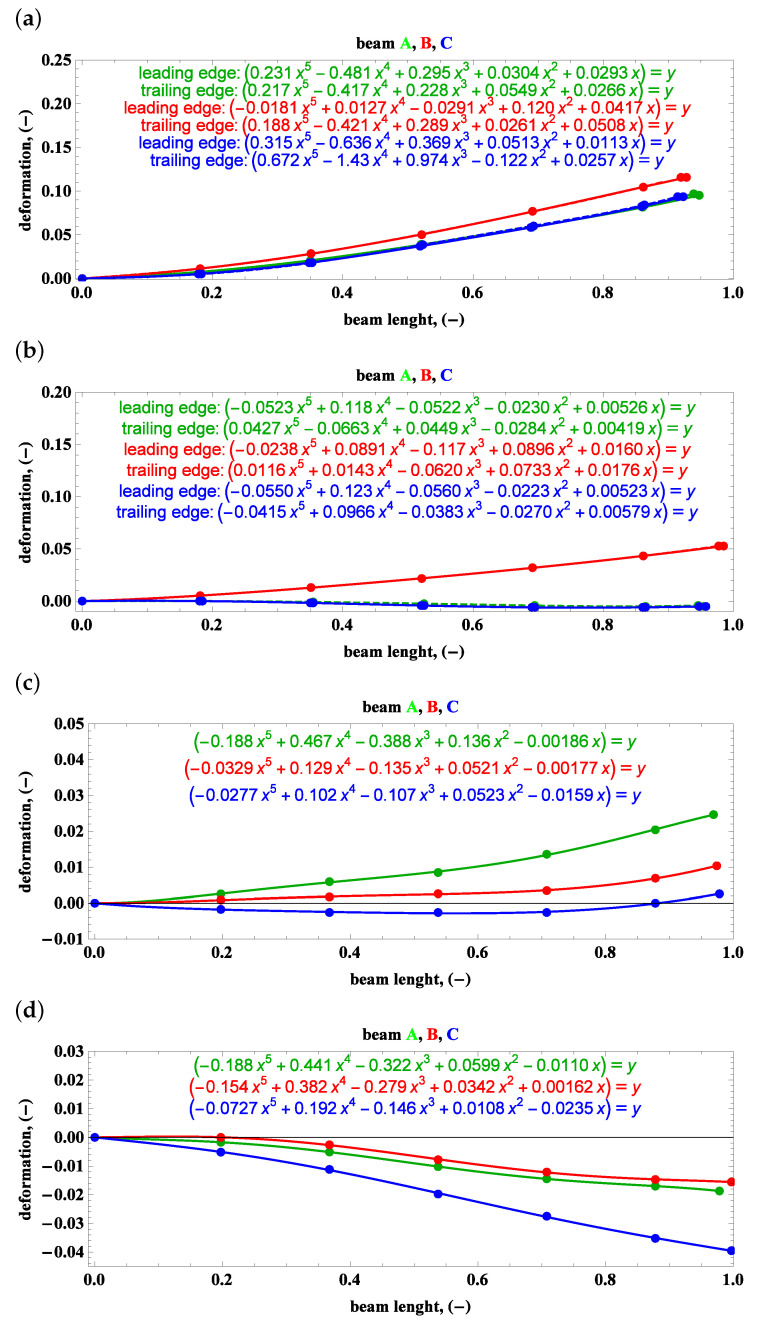
Side view—beam deformations and their description by polynomials: (**a**) preset angle $45^{\circ }$ and angular speed 100 rpm, (**b**) preset angle $45^{\circ }$ and angular speed 50 rpm, (**c**) preset angle $5^{\circ }$ and angular speed 100 rpm, (**d**) preset angle $5^{\circ }$ and angular speed 50 rpm.

**Figure 15:**
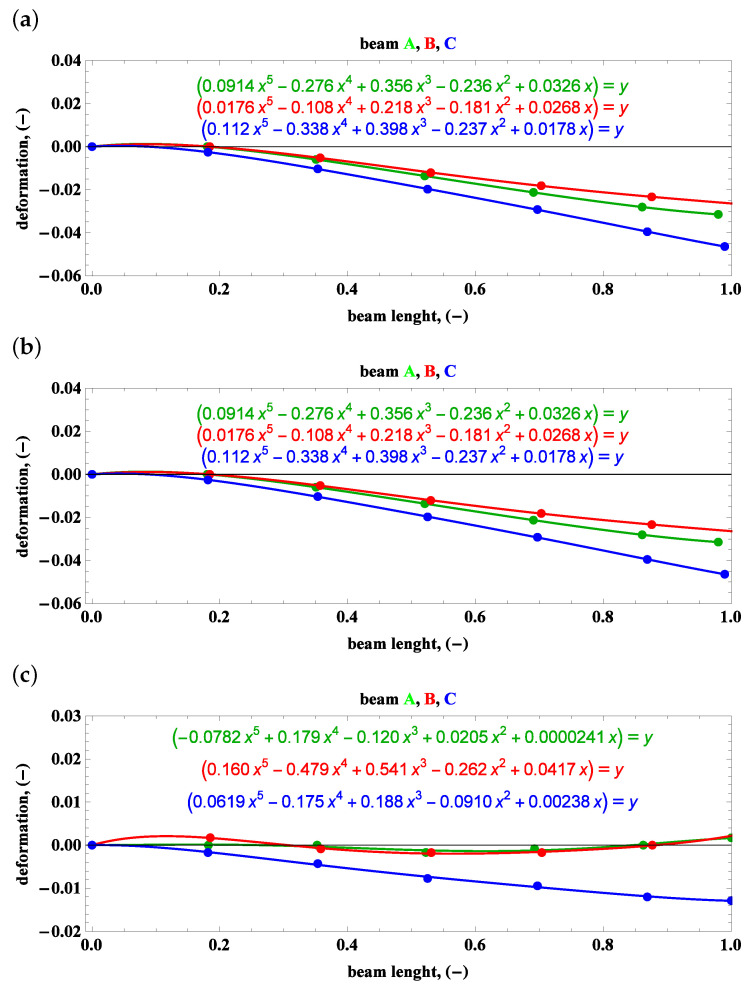
Side view—beam deformations and their description by polynomials: (**a**) preset angle $0^{\circ }$ and angular speed 50 rpm, (**b**) preset angle $0^{\circ }$ and angular speed 100 rpm, (**c**) preset angle $0^{\circ }$ and angular speed 150 rpm.

Sets of points in the plane of the beam length vs. the relative deformation are approximated by polynomials, which is the main purpose of the camera measurements gathered in Figure 13, Figure 14 and Figure 15.

## 5. Results—Time Series

In this section, time series from the strain gauges are analyzed in detail. On each beam near to the fixed end, the strain gauge is embedded, the signal from which is sent wirelessly to the software; see Figure 2. In our study, we focused on the longitudinal direction of the beams. The results are grouped according to the preset angle ($0^{\circ }$, $5^{\circ }$, $45^{\circ }$, and $90^{\circ }$).

### 5.1. Preset Angle $0^{\circ }$

**Figure 16:**
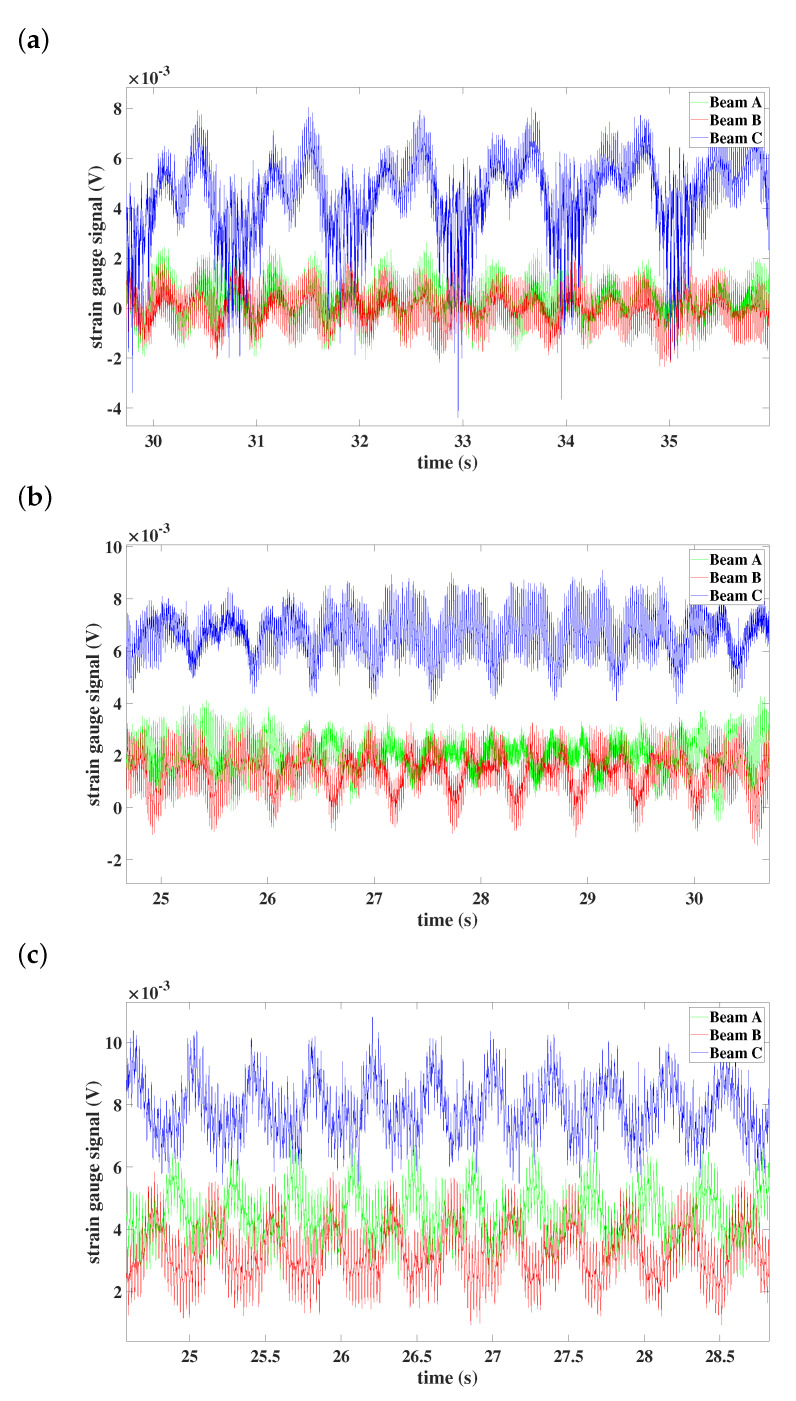
Time histories of individual beams: (**a**) preset angle $0^{\circ }$ and angular speed 50 rpm, (**b**) preset angle $0^{\circ }$ and angular speed 100 rpm, (**c**) preset angle $0^{\circ }$ and angular speed 150 rpm.

From the time series of the preset angle $0^{\circ }$—see Figure 16—one can see that vibrations are localized in beam C for every studied case of angular velocity. Moreover, at the lowest angular speed (Figure 16a), all beams are synchronized, but with an increased speed of 100 rpm, all of them vibrate differently (see Figure 16b). With the highest angular speed, the synchronization with locked phase can be observed—see Figure 16c.

### 5.2. Preset Angle $5^{\circ }$

**Figure 17:**
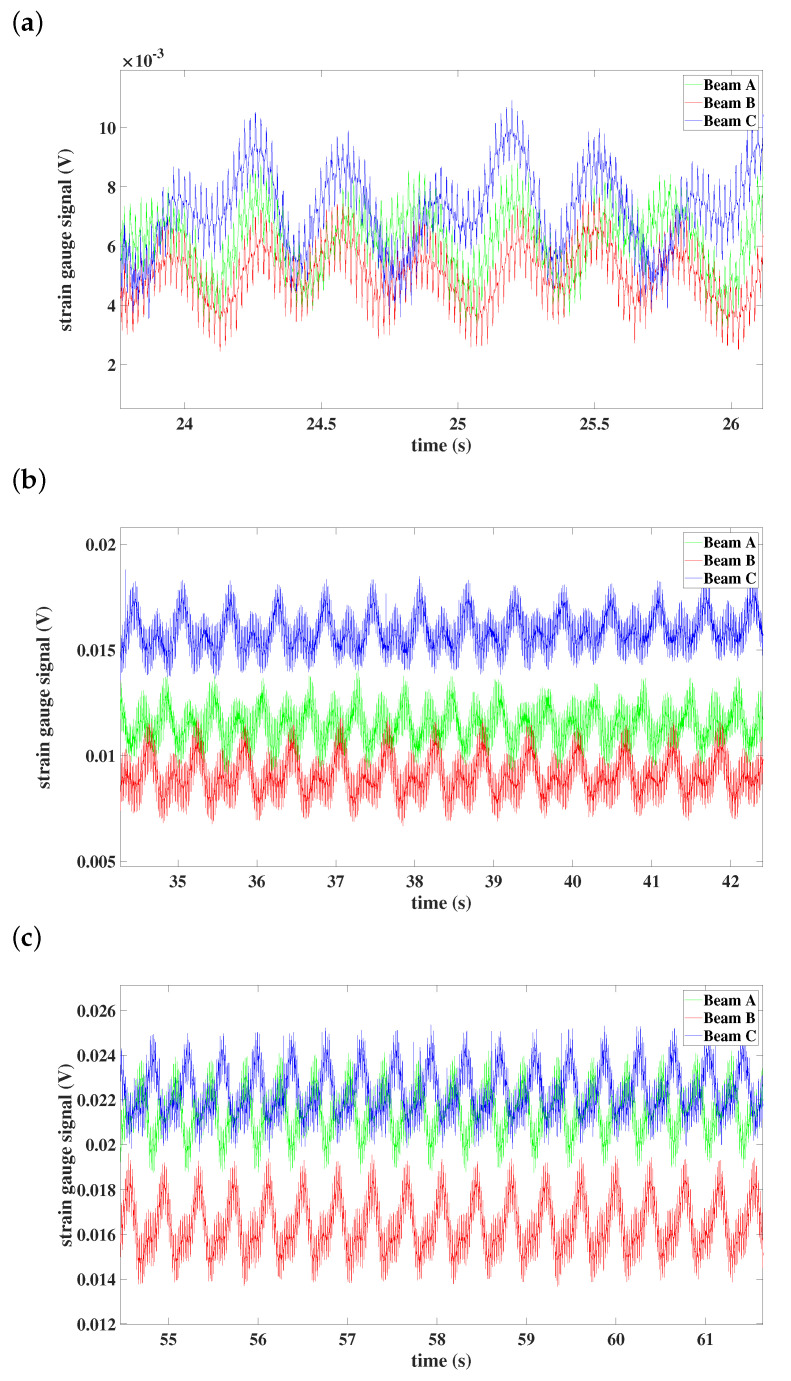
Time histories of individual beams: (**a**) preset angle $5^{\circ }$ and angular speed 50 rpm, (**b**) preset angle $5^{\circ }$ and angular speed 100 rpm, (**c**) preset angle $5^{\circ }$ and angular speed 150 rpm.

In the case of the rotor with preset angle $5^{\circ }$ of beams, the localization of the vibration changes as well as for different angular speeds—see Figure 17. When the constant angular velocity equals 50 rpm, all beams move synchronously and oscillate with similar amplitudes (Figure 17a). The synchronization between beams A and B can be observed when the system rotates with an angular speed 100 rpm; beam C is synchronized in antiphase with beams A and B (Figure 17b). The synchronization phenomenon does not change in the case of the angular speed equal to 150 rpm, but the vibrations are localized in beams A and C.

### 5.3. Preset Angle $45^{\circ }$

**Figure 18:**
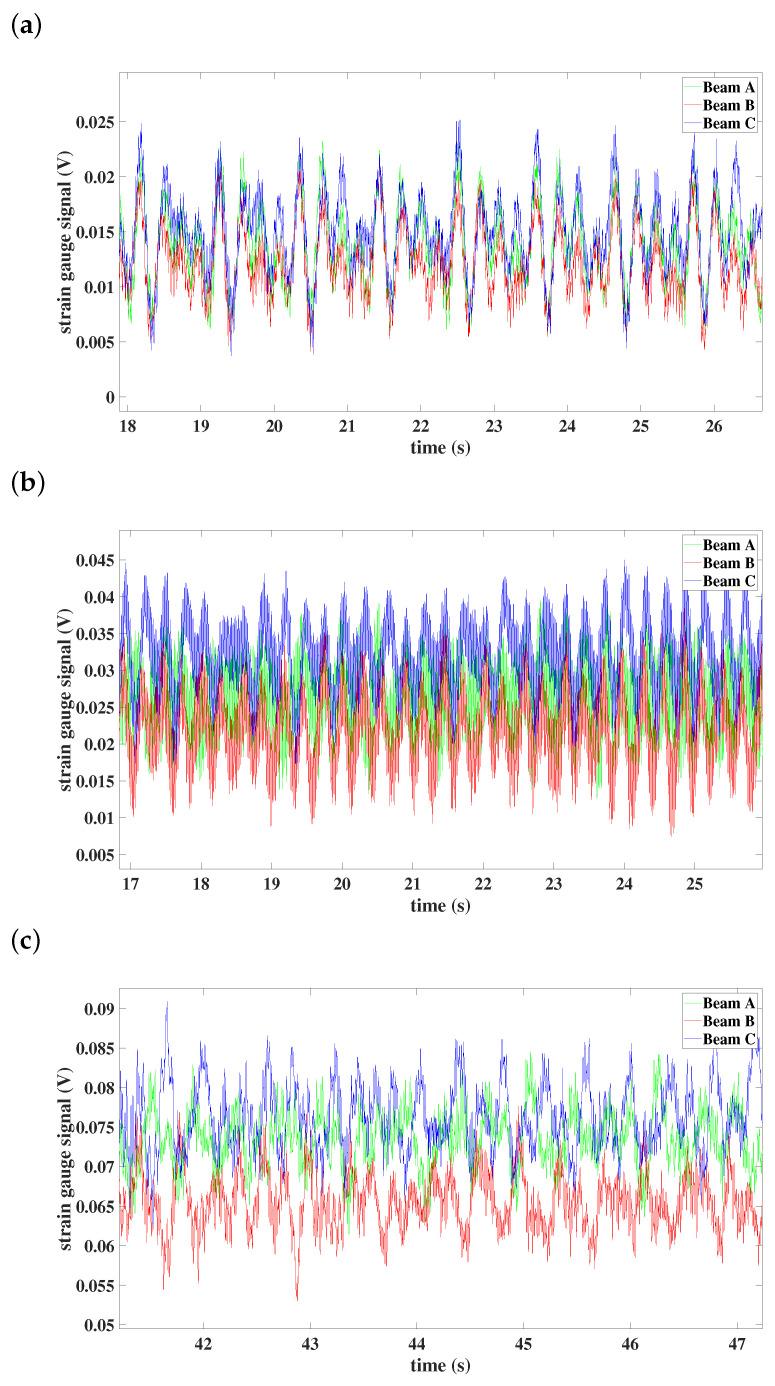
Time histories of individual beams: (**a**) preset angle $45^{\circ }$ and angular speed 50 rpm, (**b**) preset angle $45^{\circ }$ and angular speed 100 rpm, (**c**) preset angle $45^{\circ }$ and angular speed 150 rpm.

Full synchronization between all three beams can be noticed in the rotation of the model with 50 rpm and preset angle of beams $45^{\circ }$—see Figure 18a. For this setting of the rotor, all beams oscillate with similar amplitudes; only in the case of the higher angular velocity (150 rpm) does asynchronous motion appear (in Figure 18c).

### 5.4. Preset Angle $90^{\circ }$

**Figure 19:**
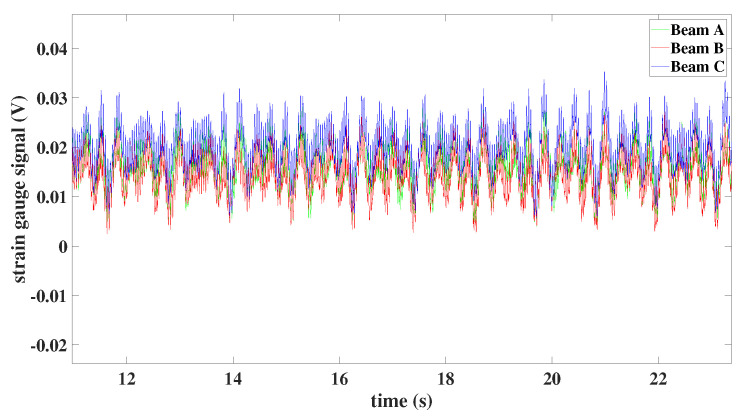
Time histories of individual beams with preset angle $90^{\circ }$ and angular speed 50 rpm.

As was mentioned before, the experimental tests of the rotor with the preset angle $90^{\circ }$ of beams created some difficulties. The DC motor power rotor was insufficient, so the tests were performed only for the angular speed equal to 50 rpm. In Figure 19, the full synchronization between all three beams can be observed and the amplitude of beams is similar.

## 6. Conclusions and Final Remarks

The paper presents experimental studies performed on a three-bladed rotor. The aerodynamic loads, as well as the synchronization phenomenon, are studied. Two types of measurements have been used—high-speed cameras and strain gauges applied to the beams. Based on the optical measurements, it is possible to notice that the zero preset angle almost does not generate lift, and drag force can be neglected. Moreover, as the angular velocity gradually grows, the centrifugal force dominates over gravity, and the beam becomes perfectly straight. Next, a positive angle of $5^{\circ }$ effectively generates lift and drag forces, and then the lift force generates the displacement of the beam end. Angular velocity of approximately 50 rpm is large enough to balance gravity, while higher angular velocities bend the beam upwards. Furthermore, based on images from the high-speed cameras, the polynomials of aerodynamic loads have been obtained. These polynomials can be used for analytical calculations of rotating structures. Additionally, the synchronization phenomenon has been studied. The full synchronization between all three beams is observed in the case of preset angles $5^{\circ }$, $45^{\circ }$, and $90^{\circ }$ with angular speed 50 rpm. Synchronization with the locked phase appears for preset angles $0^{\circ }$ and 150 rpm. Finally, in the case of preset angle $5^{\circ }$, the localization of the beams’ vibration changes dynamically for various angular velocities.
